# Obesity, altered oxidative stress, and clinical correlates in chronic schizophrenia patients

**DOI:** 10.1038/s41398-018-0303-7

**Published:** 2018-11-29

**Authors:** Huimei An, Xiangdong Du, Xingbing Huang, Lingyan Qi, Qiufang Jia, Guangzhong Yin, Chunling Xiao, Xu-Feng Huang, Yuping Ning, Ryan M Cassidy, Li Wang, Jair C. Soares, Xiang Yang Zhang

**Affiliations:** 1 Beijing Hui-Long-Guan hospital, Peking University, Beijing, China; 2 Suzhou Psychiatric Hospital, The Affiliated Guangji Hospital of Soochow University, Suzhou, Jiangsu Province China; 3 The Affiliated Brain Hospital of Guangzhou Medical University (Guangzhou Huiai Hospital), Guangzhou, China; 4 School of Medicine, Illawarra Health and Medical Research Institute, University of Wollongong, Wollongong, NSW2522 Australia; 5 Department of Psychiatry and Behavioral Sciences, The University of Texas Health Science Center at Houston, Houston, TX USA; 6 Institute of Psychology, Chinese Academy of Science, Beijing, China

## Abstract

Antipsychotic pharmacotherapy is strongly obesogenic and is associated with increased oxidative stress in patients with schizophrenia. However, whether these changes reflect psychopathology, antipsychotic efficacy, or some other factor is not known. Our study aims to investigate the degree of oxidative stress in different BMI categories and to identify clinical symptomatology that may be paired with increased oxidative stress in a schizophrenia population. To this end, we performed a cross-sectional study and recruited 89 long-term inpatients with schizophrenia and collected the following variables: plasma malondialdehyde (MDA), superoxide dismutase (SOD), catalase (CAT), and glutathione peroxidase (GPx), routine biochemical analysis, and psychopathology through the Positive and Negative Syndrome Scale (PANSS). The results indicate that the levels of the lipid peroxidation product, MDA, were significantly higher in the high BMI group than the low (normal) BMI group. As expected, high BMI was associated with an atherogenic lipid profile; however, it was also associated with fewer psychopathological symptoms. Multiple regression analysis found that MDA levels, the PANSS general psychopathology subscore, and triglyceride levels (all p < 0.05) were independent contributors to the BMI in patients. These results suggested that oxidative stress may play an important role in antipsychotic-induced weight gain. Further investigations using the longitudinal design in first-episode schizophrenia patients are needed to explore the beneficial effect of antioxidants on the abnormal lipid metabolism mediated by antipsychotic treatment.

## Introduction

Weight gain is a common side effect of antipsychotics, affecting 40–60% of schizophrenia patients^1,2^. Antipsychotic-induced weight gain is a leading cause of noncompliance, leading to increased risk for relapse^3,4^. Moreover, obesity is also linked to greater morbidity, mortality, and decreased life expectancy due to an increased risk for cardiovascular and malignant disorders^5–8^. Being obese can affect psychological well-being, leading to lower quality of life^9^.

Oxidative stress occurs when there is an overproduction of reactive oxygen species (ROS) or a deficiency of cellular antioxidant defense mechanisms^10,11^. Accumulating evidence suggests that increased oxidative stress may be involved in the pathophysiology of schizophrenia^12–14^. Several studies have shown oxidative stress in schizophrenia, including disrupted activities of antioxidant enzymes superoxide dismutase (SOD), catalase (CAT), glutathione peroxidase (GPx), and glutathione (GSH)^15–18^; increased lipid peroxidation products, thiobarbituric acid reactive substances (TBARS), and malondialdehyde (MDA)^19–22^; as well as decreased nonenzymatic antioxidants in plasma, serum, red blood cells, and cerebrospinal fluid^23,24^. Preclinical and clinical studies have suggested that both typical and atypical antipsychotic medications induce oxidative stress, particularly with long-term antipsychotic treatment^19,25–27^.

Fat accumulation and obesity are linked to enhanced oxidative stress^28,29^. Several studies have established the correlation of biomarkers and end-products (such as lipid peroxidation products) of free radical-mediated oxidative stress with body mass index (BMI) as well as an inverse relationship between BMI and biomarkers of an effective antioxidant system^30–32^. It appears that ROS themselves can act to increase adipogenesis, and alter the production of adipocytokines^28,33–35^. Thus, it appears that there is an intrinsic relationship between oxidative stress and obesity.

In view of the marked alterations of oxidative stress and higher prevalence of obesity in schizophrenia patients and the intrinsic relationship between oxidative stress and obesity, it would be important to explore the association between them in schizophrenia patients. However, to our best knowledge, no study has investigated the alterations of oxidative stress in different BMI groups of schizophrenia patients. The goal of this study was to address this gap through exploring the following: (1) whether the plasma levels or activity of oxidative stress-related markers, including SOD, CAT, GPx, and MDA were altered in different BMI groups of schizophrenia patients; (2) whether the differences of clinical symptoms and lipid profiles were observed in different BMI groups of patients; (3) whether there were relationships between oxidative stress, obesity, and clinical symptoms in this population.

## Materials and methods

### Subjects

This is a cross-sectional study. Eighty-nine inpatients with schizophrenia were recruited from Beijing Hui-Long-Guan hospital, a Beijing-city owned psychiatric hospital. All patients met the following inclusion criteria: (1) age 35–65 years, Han Chinese; (2) confirmed DSM-IV diagnosis of schizophrenia; (3) at least 5 years of illness; and (4) stable doses of oral antipsychotic drugs for at least 12 months before enrollment. The average duration of disease was 28.4 ± 8.4 years and average duration of current antipsychotic treatment was 13.3 ± 8.4 years.

Since admission, all patients received dietetically balanced hospital meals which were occasionally supplemented by gifts (usually fruit). Patients had the opportunity to exercise for about an hour per day. Antipsychotic drug treatment was mainly monotherapy, primarily clozapine and risperidone.

A complete medical history, physical examination, and laboratory tests were obtained from patients. Any subjects with major medical illness were excluded. None of the subjects met the criteria for drug or alcohol abuse or dependence. All patients who received any immunomodulators or antioxidants in the last 12 weeks were excluded from the study. All subjects gave informed consent to participate in the study, which was approved by the Institutional Review Board of Beijing HuiLongGuan Hospital.

### BMI measurements

Bodyweight and height were assessed in a standardized fashion to calculate BMI (weight over squared height, kg/m^2^). Height was measured to the nearest millimeter, with the subjects barefooted and standing upright. Bodyweight was measured with an electronic scale calibrated to ± 0.1 kg; subjects were weighed in light indoor clothing.

According to the Chinese Working Group on Obesity in China (WGOC) criteria^36^, patients were defined as obese with BMI ≥ 28 kg/m^2^ and overweight with 24 ≤ BMI < 28 kg/m^2^. Hence, our patients were classified as low BMI group (BMI≤24 kg/m^2^) and high BMI group (BMI > 24 kg/m^2^).

### Clinical measurements

The patients’ psychopathology was assessed using the Positive and Negative Syndrome Scale (PANSS) by two psychiatrists, who had simultaneously attended a training session on using PANSS. Repeated assessments for the PANSS total score maintained an inter-rater correlation coefficient greater than 0.8.

### Routine biochemical analysis

Venous blood from the forearm vein was collected between 7 and 9 AM following an overnight fast. The biochemical analysis was performed by a technician blind to the clinical status of the subjects. The serum lipid profiles, including triglycerides (TG), total cholesterol (TC), high-density lipoprotein cholesterol (HDL-C), low-density lipoprotein cholesterol (LDL-C), and cholesterol (CHO), apolipoprotein (ApoA1) and ApoB were measured in the hospital laboratory center using commercially available kits from Leadman (Beijing Leadman Biotechnology Co. Ltd., Beijing, China) and by an automatic biochemistry analyzer AU2700 (Olympus, Japan).

### Oxidative stress assessment

Blood samples from schizophrenia inpatients were collected between 7 and 9 AM following an overnight fast. The plasma was separated, aliquoted, and stored at −70 °C before use. All antioxidant enzymes and lipid peroxidation products in plasma were measured by a technician, who was blind to the clinical status of subjects.

### Determination of lipid peroxidation

Lipid peroxidation levels were monitored by determining the end product of lipid peroxidation MDA by the thiobarbituric acid (TBA) method, which was modified from the method of Yagi^37^. Plasma MDA values were calculated using the extinction coefficient of MDA–thiobarbituric acid complex at 532 nm. MDA results were expressed as nmol/ml.

### SOD activity measurement

Determinations of plasma total SOD activities were performed using a standard assay involving spectrophotometric determination of the inhibition of superoxide-induced formation of nitrite from hydroxylamine, as described by Oyanagui^38^. Xanthine–xanthine oxidase provided the superoxide source. One unit is defined as the amount of SOD that inhibits 50% of nitrite formation under the assay conditions. Activity was expressed as units per milliliter plasma (U/ml). The inter- and intra-assay coefficient of variation for SOD activity was 4.1% (*n* = 6) and 3.2% (*n* = 6), respectively.

### GPx activity measurement

GPx activity was measured by a modification of the method reported in previous study^39^. The enzymatic reaction was initiated by adding H_2_O_2_ to the reaction mixture containing reduced GSH, reduced nicotinamide adenine dinucleotidephosphate (NADPH), and glutathione reductase. The change in absorbance at 340 nm was monitored by a spectrophotometer. One unit of GPx is defined as micromoles of NADPH oxidized per minute. Activity was given in units per liter plasma volume. The intra- and inter-assay coefficient of variation was 4.8% (*n* = 6) and 5.7% (*n* = 6), respectively.

### CAT activity measurement

CAT activity was assayed by the method of Aebi^40^. This method was based on the decomposition of hydrogen peroxide by CAT. CAT catalyzes the transformation of hydrogen peroxide to water and oxygen. CAT activity was determined by monitoring the decreased absorbance spectrophotometrically at 240 nm due to degradation of hydrogen peroxide. One unit of CAT was defined as the amount of enzyme that decomposes 1 μmol H_2_O_2_/min under specific conditions. CAT activity is expressed as U/ml. The intra- and inter-assay coefficient of variation was 4.5% (*n* = 6) and 5.9% (*n* = 6), respectively.

### Statistical analysis

Demographic and clinical variables of different BMI groups were compared using analysis of variance (ANOVA) for continuous variables and chi-squared test for categorical variables. Since the majority of the variables were normally distributed in different BMI groups (Shapiro–Wilk test), the principal outcome analysis consisted of analysis of variance for comparison between two different BMI groups. When significance was found in ANOVA, the effects of gender, age, education, duration of illness, antipsychotic treatment (type, dose, and duration of treatment), and the PANSS total and its subscale scores were tested by adding these variables to the analysis model as covariates. Relationships between variables were assessed with Pearson’s product moment correlation coefficients. Bonferroni corrections were applied to each test to adjust for multiple testing. Multivariate regression analysis (stepwise regression model) was used to assess correlations of BMI and oxidative stress while adjusting for various potentially confounding variables of gender, age, education, duration of illness, antipsychotic treatment (type, dose, and duration of treatment), and the PANSS total and its subscale scores. SPSS version 16.0 was used to do all statistical analyses. Statistical significance was defined as *P* < 0.05.

## Results

### Demographics

Demographic data and lipid profiles in the two BMI groups are displayed in Table 1. There was no significant difference in age, education, gender, duration of treatment, age of onset, hospitalization numbers, or daily antipsychotic dose (chlorpromazine equivalent) and the type of antipsychotic drugs between the two different BMI groups (all *P* > 0.05). However, TG levels (F = 6.557, df = 1,86, *P* = 0.012) and LDL-c (F = 4.317, df = 1,86, *P* = 0.041) were significantly higher in the high BMI group than the low BMI group. Moreover, correlation analysis showed that BMI was positively correlated with TG (r = 0.438, *P* = 0.000) and LDL-c (r = 0.245, *P* = 0.021), respectively.

**Table 1:** Demographic data and lipid profiles in different BMI groups

Variable	Low BMI group (*n* = 34)	High BMI group (*n* = 55)	F or χ^2^	df	*P*-value
Age (years)	51.2 ± 6.8	52.1 ± 8.7	0.272	1,87	0.603
Education (years)	9.6 ± 2.5	9.8 ± 2.5	0.157	1,87	0.693
Male/female	26/8	34/21	2.054	1	0.171
Duration of illness (years)	27.9 ± 8.7	28.3 ± 9.5	0.054	1,87	0.816
Age of onset (years)	23.6 ± 5.7	23.6 ± 6.2	0.000	1,87	0.989
Hospitalization numbers	3.8 ± 1.9	4.0 ± 3.0	0.150	1,87	0.699
BMI (kg/m^2^)	21.1 ± 2.3	27.0 ± 2.2	144.9	1,87	0.000
Daily AP dose (mg) (CPZ equivalent)	338.6 ± 133.3	315.4 ± 151.0	0.541	1,87	0.464
Risperidone/clozapine	12/22	10/45	3.306	1	0.081
TG	1.4 ± 0.7	1.9 ± 1.1	6.557	1,86	0.012
HDL-c	1.1 ± 0.1	1.1 ± 0.2	2.384	1,86	0.126
LDL-c	2.8 ± 0.6	3.1 ± 0.6	4.317	1,86	0.041
APOA	1.2 ± 0.1	1.2 ± 0.1	1.370	1,86	0.245
APOB	0.7 ± 0.1	0.8 ± 0.5	3.467	1,86	0.066
CHO	4.3 ± 0.8	4.6 ± 1.1	1.942	1,86	0.167

*AP* antipsychotic, *CPZ* chlorpromazine, *BMI* body mass index, *TG* triglycerides, *TC* total cholesterol, *HDL-c* high-density lipoprotein cholesterol, *LDL-c* low-density lipoprotein cholesterol, *CHO* cholesterol

### MDA levels and antioxidant enzyme activities in different BMI groups

**Fig. 1:**
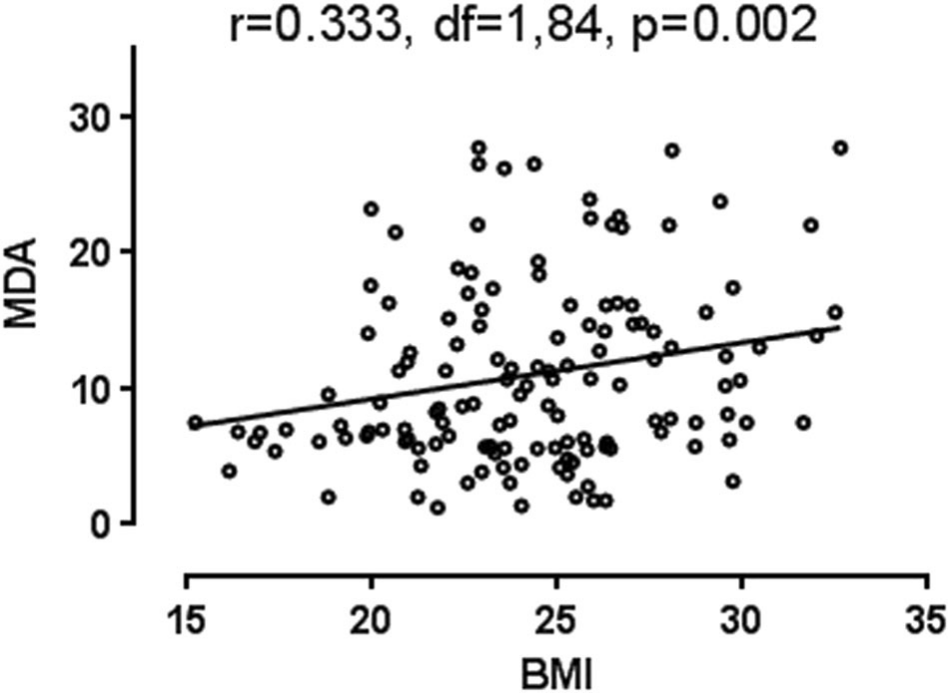
Correlation analysis showed that MDA levels were positively associated with BMI

Plasma MDA levels were significantly higher in the high BMI group than the low BMI group (F = 7.472, df = 1,84, *P* = 0.008). This difference remained significant after covarying for gender, age, education, duration of illness, and daily CPZ equivalent dose (*P* = 0.015). Correlation analysis showed that MDA levels were positively associated with BMI (r = 0.333, df = 1,84, *P* = 0.002) (Fig. 1). However, levels of SOD, GPx, and CAT did not differ between the two groups, indicating that this finding is specific to MDA (Table 2).

**Table 2:** Antioxidant enzymes and MDA levels in different BMI groups

Variable	Low BMI group	High BMI group	F	df	*P*-value
SOD	86.9 ± 18.8	82.9 ± 17.7	1.014	1,86	0.317
GPx	104.2 ± 30.2	100.9 ± 25.2	0.351	1,84	0.576
CAT	2.1 ± 1.5	1.6 ± 1.1	2.221	1,85	0.140
MDA	8.4 ± 5.0	12.6 ± 7.6	7.472	1,84	0.008

*BMI* body mass index, *SOD* superoxide dismutase, *CAT* catalase, *GPx* glutathione peroxidase, *MDA* malondialdehyde

### Clinical symptoms in different BMI groups

As shown in Table 3, the PANSS total score (F = 6.232, df = 1,84, *P* = 0.015), negative (F = 5.019, df = 1,84, *P* = 0.028) and general psychopathology subscore (F = 4.766, df = 1,84, *P* = 0.032) were significantly lower in the high BMI group than the low BMI group. The positive symptom subscore did not significantly differ between groups (*P* = 0.14). When gender, age, education, duration of illness, and daily CPZ equivalent dose were added as covariates, the differences in the PANSS total score, negative and general psychopathology subscore remained significant (all *P* < 0.05). Furthermore, due to an imbalanced number of the patients treated with clozapine between the high and low BMI groups (85% vs. 65%), we further did ANCOVA using the antipsychotic types (clozapine vs. non-clozapine or clozapine vs. risperidone) as covariates, still showing the significant differences in the PANSS total score, negative and general psychopathology subscore between the low and high BMI groups (all *P* < 0.05). Correlation analysis further showed that negative psychopathology subscore was negatively associated with BMI (r = –0.243, df = 1,84, *P* = 0.026).

**Table 3:** Clinical symptoms in different BMI groups

Variable	Low BMI group	High BMI group	F	df	*P*-value
PANSS total score	75.1 ± 17.8	66.4 ± 14.1	6.232	1,84	0.015
P subscore	16.7 ± 6.1	14.9 ± 5.3	2.202	1,84	0.142
N subscore	26.0 ± 6.0	23.3 ± 4.9	5.019	1,84	0.028
G subscore	35.6 ± 9.4	31.7 ± 7.1	4.766	1,84	0.032

*PANSS* positive and negative syndrome scale, *P* positive symptom, *N* negative symptom, *G* general psychopathology syndrome

### Relationship between BMI, oxidative stress, clinical symptoms, and lipid profiles

Using correlation analysis, we found that GPx activity was negatively associated with TG (r = –0.299, *P* = 0.005) and SOD activity was positively correlated with the negative psychopathology subscore (r = 0.330, *P* = 0.002) and the PANSS total score (r = 0.229, *P* = 0.037). Multiple regression analysis showed that MDA levels (β = 0.245, t = 2.490, and *P* = 0.015), the PANSS general psychopathology subscale (β = −0.207, t = −2.129, and *P* = 0.037), and TG levels (β = 0.434, t = 4.433, and *P* = 0.000) were independent contributors to the BMI after controlling for gender, age, education, duration of illness, and daily CPZ equivalent dose.

## Discussion

The major findings of the present study on schizophrenia long-term inpatients are the following: (1) the levels of the lipid peroxidation product MDA were significantly higher in the high BMI group; (2) the high BMI group showed fewer psychopathological symptoms and a more atherogenic lipid profile; (3) BMI status, oxidative stress, lipid profiles, and clinical symptoms were tightly correlated.

We found that the lipid peroxidation product MDA levels were significantly higher in the high BMI group and that MDA levels positively associated with BMI. Also, we found a negative association between GPx activity and TG levels. Our results were consistent with previous studies showing a close correlation between BMI and the biomarkers and end-products of free radicals-mediated oxidative stress^31,32,41^, as well as an inverse association between obesity and antioxidant defense markers^30^. Previous studies showed a correlation of fat accumulation with the overproduction of ROS^28^ and that obesity is associated with excessive ROS production and activation of the antioxidant defense system. This suggests that metabolic disease may be an important contributor to systemic oxidative stress^35^. Conversely, chronic elevation in ROS has been found to promote insulin resistance and adipogenesis, suppress adiponectin expression and secretion, and upregulate the expression of proinflammatory cytokines (PAI-1, IL-6) and macrophage chemoattractive molecule (MCP-1)^28,42^. It is possible that increased ROS lead to dysfunction of lipid metabolic pathways through direct lipid peroxidation^35^. Clinical studies have also found decreased activities of SOD, GPX, and CAT and increased lipid peroxidation products, including MDA, lipid hydroperoxides, and conjugated dienes in the plasma of obese subjects^32,43–45^, providing evidence for this theory.

In schizophrenia, antipsychotic treatment induces obesity and is associated with the alteration of oxidative stress parameters^19,25–27^. It may be the case that primary oxidative damage induced by antipsychotics then leads to the development of obesity. However, this is only our speculation as the exact relationship between overweight/obesity and enhanced oxidative stress in the chronic schizophrenia patients warrants future investigation. In either case, elevated ROS are independently associated with poorer health outcome and should be addressed; use of antioxidants such as EGB761 combined with antipsychotic drugs targeting may reduce oxidative stress and potentially alleviate clinical symptomatology^46,47^, especially for those with higher BMI. Further investigations are needed to elucidate the beneficial effect of antioxidants on the outcome of schizophrenia, especially for those obese schizophrenia patients.

Our study demonstrated that the high BMI group experienced fewer psychopathological symptoms. The results were consistent with previous studies showing that antipsychotic-induced weight gain was associated with improvement in psychopathology^48,49^. We also have previously demonstrated an inverse correlation between BMI and PANSS total score in schizophrenia patients treated with long-term clozapine^50^ and between BMI and the PANSS negative symptom score in chronic and medicated schizophrenia patients^2^. The improvement of the psychopathology accompanied by weight gain may be a side effect indicating antipsychotic medication efficacy^48^. It is worthy of mentioning that the majority of the high BMI group (85%) but the minority of the low BMI group (65%) were treated with clozapine. Since clozapine was more effective but caused more weight gain than risperidone, it is likely that the patients on more clozapine showed higher BMI and lower PANSS scores than those on risperidone. However, after the ANCOVA test using the antipsychotic types (clozapine vs. non-clozapine or clozapine vs. risperidone) as covariates, the significant differences in the PANSS scores still remained, suggesting that the relationship between the lower PANSS scores and high BMI group might not be due to the effects of the antipsychotic medications. However, several other studies reported that the weight gain or obesity was not significantly associated with changes in symptoms in response to antipsychotic treatment^51,52^. The possible reasons for this difference may be related to some complicating factors, such as type, dose, treatment duration, medication adherence, clinical status, the baseline level of psychopathology, reliability of assessment, and concomitant medications^48,51^.

We also demonstrated that SOD activity was positively correlated with the negative psychopathology subscore and the PANSS total score in chronic schizophrenia patients. This result is consistent with work showing a similar association in the first few weeks of antipsychotic therapy^26^. Our own previous study also reported that greater change in SOD was correlated with greater symptom improvement during antipsychotic treatment^27^. These results suggested that antioxidant enzyme SOD activity might be associated with psychopathological symptoms severity. However, Tsai et al. also reported the seemingly contradictory result that PANSS total scores were significantly *negatively* correlated with serum GPx activity and GSH levels ^26^. The exact mechanisms underlying the association between the psychopathological symptoms and oxidative stress parameters are still unclear.

We also demonstrated that the high BMI group had higher atherogenic lipid profiles in chronic schizophrenia patients which is entirely consistent with current medical knowledge on the relationship between obesity and dyslipidemia^53–55^.

Several limitations of the study should be noted here. First, this was a cross-sectional study design and cannot show direct causality between weight gain, altered oxidative stress biomarkers, and clinical symptoms in schizophrenia patients. Second, the sample consisted of long-term inpatients, with more baseline-severe psychopathology, longer duration of illness, treatment, different medication history, and hospitalization than typical schizophrenia patients. Measuring the variable reported in this study in a longitudinal cohort design with first-episode and medication-naive patients could clarify the relationships between oxidative stress, weight gain, and clinical responses. Third, previous studies have shown that clozapine or risperidone treatment have different antioxidant effects^56,57^. This fact is an important confounding factor of this study. Moreover, although all patients received either risperidone or clozapine monotherapy when they were recruited, we did not collect the data for the medications the patients had taken before risperidone or clozapine. Thus, whether or how the medication history may contribute to the alterations of oxidative stress parameters in our current study is still unknown. Therefore, it is important to take into account the type of antipsychotic medications in the future study of oxidative stress. Fourth, in this study, TBARS assay was used to measure the plasma MDA values, which is an imprecise and nonspecific measure of MDA^58^. One alternative approach is to separate interfering substances from MDA–TBA adduct by high-pressure liquid chromatography (HPLC) prior to spectrophotometric measurement. This HPLC procedure provides considerably better sensitivity and specificity, which results in more reliable reference values than any previously published results. Another limitation of our study is that we did not assess smoking status, although smoking is highly prevalent in this patient population—over 75% as found in our previous study^59^. This factor may affect weight gain, oxidative status, and symptomatology. However, given that nicotine is a well-known appetite suppressant and appears to reduce negative symptomatology^59^, the correlation would not be in the direction demonstrated in our current work.

In conclusion, we have demonstrated that high BMI is associated with the increased oxidative stress, higher atherogenic lipid profiles, and lower psychopathological symptoms in chronic schizophrenia patients. Due to the cross-sectional design, we cannot demonstrate causality between BMI, oxidative stress, and clinical psychopathological symptoms in our schizophrenia patients. However, it appears that oxidative stress may play an important role in antipsychotic-induced weight gain. Therefore, we speculate that combining the antioxidants with antipsychotic drugs may reduce oxidative stress and further may reduce or prevent the antipsychotic-induced weight gain in schizophrenia patients. Further investigations using the longitudinal design in first-episode schizophrenia patients are needed to explore the beneficial effect of antioxidants on the therapeutic outcome, as well as on the abnormal lipid metabolism mediated by antipsychotic treatment.
